# Identification of Amazonian Trees with DNA Barcodes

**DOI:** 10.1371/journal.pone.0007483

**Published:** 2009-10-16

**Authors:** Mailyn Adriana Gonzalez, Christopher Baraloto, Julien Engel, Scott A. Mori, Pascal Pétronelli, Bernard Riéra, Aurélien Roger, Christophe Thébaud, Jérôme Chave

**Affiliations:** 1 Laboratoire Evolution et Diversité Biologique, Université Paul Sabatier and CNRS, UMR 5174, Toulouse, France; 2 INRA, UMR Ecologie des Forêts de Guyane, Kourou, French Guiana, France; 3 Institute of Systematic Botany, New York Botanical Garden, Bronx, New York, United States of America; 4 CIRAD, UMR Ecologie des Forêts de Guyane, Kourou, French Guiana, France; 5 Laboratoire Fonctionnement, Evolution et Mécanismes Régulateurs des Ecosystèmes Forestiers, CNRS, UMR 5176, Brunoy, France; University of Zurich, Switzerland

## Abstract

**Background:**

Large-scale plant diversity inventories are critical to develop informed conservation strategies. However, the workload required for classic taxonomic surveys remains high and is particularly problematic for megadiverse tropical forests.

**Methodology/Principal Findings:**

Based on a comprehensive census of all trees in two hectares of a tropical forest in French Guiana, we examined whether plant DNA barcoding could contribute to increasing the quality and the pace of tropical plant biodiversity surveys. Of the eight plant DNA markers we tested (*rbcLa, rpoC1, rpoB, matK, ycf5, trnL, psbA-trnH, ITS*), *matK* and *ITS* had a low rate of sequencing success. More critically, none of the plastid markers achieved a rate of correct plant identification greater than 70%, either alone or combined. The performance of all barcoding markers was noticeably low in few species-rich clades, such as the Laureae, and the Sapotaceae. A field test of the approach enabled us to detect 130 molecular operational taxonomic units in a sample of 252 juvenile trees. Including molecular markers increased the identification rate of juveniles from 72% (morphology alone) to 96% (morphology and molecular) of the individuals assigned to a known tree taxon.

**Conclusion/Significance:**

We conclude that while DNA barcoding is an invaluable tool for detecting errors in identifications and for identifying plants at juvenile stages, its limited ability to identify collections will constrain the practical implementation of DNA-based tropical plant biodiversity programs.

## Introduction

The Neotropics hold an estimated 78,800 flowering plant species, over a third of the world's total [1]. Yet, tropical forests are being degraded at a fast pace [2], [3], and over half of the estimated 11,000 Amazonian tree species may face a direct risk of extinction [4]. Thus large-scale biodiversity inventories are critically needed in order to develop informed conservation strategies for these diverse ecosystems [5], [6]. Significant progress in mapping the distribution of Neotropical plants has been achieved over the past decades [7]–[11], but many areas are still under-collected and species identification remains a challenging task in many plant families. An example was recently provided by Pitman *et al.* (2008), who conducted a tree species diversity survey along a 700-km transect that cuts across one of the most diverse parts of the Amazon, between Ecuador and Brazil [12]. Based on traditional botanical sampling, they were able to identify 97% of the sampled stems to the genus, and counted a total of 435 tree genera. Yet, in their statistical analyses, they decided to conservatively exclude the genera that were difficult to identify in the field when only sterile material was available. Their choice of excluding no less than 20.7% of the genera, and 15.7% of the sampled stems resulted in loss of information, the influence of which on their conclusions is unknown.

With the advent of high-throughput DNA sequencing, it has been suggested that universally amplified, short, and highly variable DNA markers (DNA barcodes) may help identify organisms to species with a high confidence, which would be useful in a wide array of applications, including biodiversity surveys [13]–[15]. DNA barcodes should be both variable enough to discriminate among closely related species and yet possess highly conserved regions so as to be easily sequenced with standard protocols. The mitochondrial marker *cytochrome c oxidase I* (CO1) has met with some success for animal groups [13], [16], but see [17]–[19]. In plants, the search for suitable genomic regions has proven more challenging. Several regions in the plastid genome (e.g. *rbcL, rpoC1, rpoB, ycf5, psbA-trnH, trnL, atpF-atpH, psbK-psbI*) as well as the internal transcribed spacer (*ITS*) of the ribosomal nuclear DNA have emerged as good candidates for plant DNA barcoding [20]–[27]. A consensus has recently emerged among the members of the Consortium for the Barcoding of Life (CBoL) Plant Working Group for using only two of these markers to barcode land plants, namely *rbcL* and *matK* [28], yet these authors point out that this combination will lead to a species-level identification in 72% of the cases only, and this resolution is unlikely to be evenly distributed across land plant species.

Echoing Chase *et al.* (2007) [29], the CBoL Plant Working Group pointed out that plant DNA barcoding should be useful in discriminating among forest seedlings, or undertaking large-scale biodiversity surveys in situations where taxonomic expertise is limiting. Yet, we are unaware of any application in this research area thus far, and the present work fills this gap. Tropical plants present challenges to DNA barcoding that are much more pronounced than those encountered when barcoding temperate plants, and today applications of plant DNA barcoding in the tropics is still unchartered land (the only exceptions being applications on genus *Compsoneura* in the Myristicaceae, see Newmaster *et al.* 2008; genus *Inga* in the Fabaceae [30]; and the orchid family [26]). DNA extraction is expected to be more difficult in tropical plants, due to the greater abundance of secondary metabolites [31], and this may compromise the overall performance of DNA barcoding [32]. In addition, the rate of lineage diversification is often high in the tropics, leading to the frequent occurrence of explosive radiations [33]–[34]. For recent lineages with great numbers of species, we thus expect that DNA barcoding will be less efficient, because species will tend to have lots of close relatives, reducing levels of interspecific divergence, as recently confirmed in genus *Inga* [35], and as should be expected in other groups [36]. Finally, it has been shown that woody plant lineages show consistently lower rates of molecular evolution as compared with herbaceous plant lineages [37], suggesting the application of DNA barcoding concepts should be more difficult for tree floras than for non-woody floras [26], [38].

In the present study, we use a plot-based sampling strategy to test the applicability of the currently proposed DNA barcoding scheme. Specifically, we examine if consensus barcodes are sufficiently variable and universal to reliably identify co-occurring Amazonian tree species, and we implement this scheme to the identification of tropical juvenile plants.

## Materials and Methods

### Study site and sampling

This study was conducted at the Nouragues Research Station, central French Guiana, in pristine lowland tropical rainforest (4°05 N, 52°40 W; [39]). Rainfall is 2824 mm y−1 (average 1988–2008) with a dry season averaging 2.5 months, from late August to early November, and a shorter dry season in March. The plant diversity of this area is high, with a local flora exceeding 1700 angiosperm species.

We sampled all trees ≥10 cm of diameter at breast height (dbh) in two 1-ha plots. Large trees were sampled by professional tree climbers while smaller trees (less than 35 cm dbh) were collected using French climbing spikes (Fonderies Lacoste, Excideuil, France, [40], [41]). A total of 1073 trees were sampled in the two plots. Voucher specimens were matched against the reference vouchers available at the Herbier de Guyane, Cayenne (CAY), and they were deposited there. Of the 301 tree morphospecies, 254 could be matched to a reference voucher with an accepted species name (94% of the inventoried individuals). These encompassed 143 genera, and 54 angiosperm families, and they spanned the most common woody plant families in Amazonia (Table S1). Individuals from the most taxonomically difficult families, such as Lauraceae, Myrtaceae, Elaeocarpaceae (*Sloanea*), or Sapotaceae (*Pouteria*), were kept into morphospecies.

For each sampled plant, we collected 1–10 cm2 of leaf tissue. Samples collected for DNA analysis were stored in 10 g of silica gel. We also collected ca. 1 cm2 of cambium tissue using a leather punch of 1 cm in diameter to test whether DNA could be extracted efficiently from this tissue [42]. Total DNA extraction was of comparable concentration with cambium and leaf tissue (results not shown), and both were used for sequencing.

### *DNA extraction and sequencing*

Up to 30 mg of dry material was ground for 2 min in a TissueLyser mixer-mill disruptor (Qiagen, California, USA) using tungsten beads. Lysis incubation was carried out at 65°C during 2 hr for cambium tissue and 1 hr for leaf tissue using CTAB 1% PVP buffer. Total DNA extraction was performed using a Biosprint 15 workstation (Qiagen, CA) following the manufacturer's protocols.

PCR amplification was performed for the coding plastid regions *rbcLa* (first part of the *rbcL* gene), *rpoC1, rpoB*, *matK*, *ycf5*, the non-coding regions *trnL* and *psbA-trnH*, and the nuclear region *ITS*. The PCR reaction mix included 0.2 µl of GoTaq® 51 U/µl (Promega), 10 µl of 5× buffer, 1 µl of 20 µM for each primer, 1 µl of dNTP 10 mM, 1 µl of DNA template and H2O for a final volume of 50 µl. For primer combinations, PCR thermal conditions, and references, see Table S2.

PCR products were purified with a MinElute PCR Purification Kit (Qiagen, CA). Cycle sequencing reactions were performed in 10 µl reactions using 1 µl of BigDye® Terminator cycle sequencing chemistry (v3.1; ABI; Warrington, Cheshire, UK) and run on ABI sequencers. The markers were sequenced in both directions. DNA fragments were visually inspected and assembled with SequencherTM 4.8 (Gene Codes Corp., Ann Arbor, Michigan, USA). In about 10% of the cases, the marker *psbA-trnH* proved difficult to sequence from the 3′ end (*trnH*), due to long poly-A and poly-T regions [43]. If and only if the single strand had a high-quality read, a single direction sequence was used. All of the sequences are deposited on GenBank (see Table S1 for the accession numbers).

We did not sequence all 1073 individuals for all candidate markers, but selected 285 individuals so as to represent all the taxonomic groups, and facilitate interspecific and congeneric comparisons. In a few markers, we increased the sequencing effort (*rbcL*, *rpoC1*, and *psbA-trnH*).

### Test of the barcoding approach on tropical saplings

Having assembled a large database of plant DNA barcodes for tropical tree species, we tested whether it could be used to identify juveniles in the same plots, which often lack the morphological characters used to identify mature plants [29]. We established two 4×4 m sapling plots within each of our two tree plots. All woody plants above 30 cm in height and <1 cm dbh (n = 252) were marked, measured, and mapped. Because it is often difficult to tell apart tree, shrub and liana saplings, we included all woody plants within the size limits, and subsequently used our identifications to infer the life form of these individuals. Based on morphology, 27% of the individuals could be reliably identified to the species, another 45% could be assigned to a clear morphotype, and 11% could be assigned to a known genus.

### Data analyses

We tested if the species were retrieved as monophyletic group with the different markers. The sequences were aligned using ClustalX version 2.0.11 with default parameters [44], and alignments were visually inspected. For each marker, we generated neighbour-joigning (NJ) trees based on sequence divergence estimated with Kimura's 2-parameter (K2P) nucleotide evolution model [45], using ClustalX and the software Mega 4.0 [46]. Node support was assessed via 1000 bootstrap replicates. Trees were also constructed for each coding marker using PhyML [47] using the most general time-reversible model of nucleotide evolution with Gamma distributed errors on mutation rates (GTR+G). In PhyML, node support was estimated using the approximate likelihood-ratio test (alrt), a much faster method for estimating branch support than either the bootstrap or Bayesian posterior probabilities [48]. We present results based on NJ and ML trees only because this has the greatest potential for computationally intensive analyses based on large datasets and other studies have shown that the choice of the phylogeny reconstruction algorithm did not significantly alter the tests of DNA barcode performance [19], [26]. In preliminary runs, we discovered that the performance of all plastid markers in recovering species as monophyletic was poor in two important groups that are easily recognized in the field: the Sapotaceae [49], and the Laureae clade in Lauraceae [50]. We then also computed the fraction of supported clades, excluding these two groups. We assumed that clades were supported when the bootstrap values exceeded 70%, or when the alrt values exceeded 80%.

Assessing monophyly using DNA barcodes has been criticized because it assumes that tree reconstruction is reliable, and that the minimal threshold on support value is a reliable criterion for clade support. Meier *et al.* (2006) have proposed an alternative criterion (‘best close match’) [17]. A threshold T is computed below which 95% of all intraspecific distances are found. If a query sequence had no match below T, it is left unidentified. Otherwise, if all matches of the query sequence are conspecific, the barcode assignment is considered to be correct. If the matches of the query sequence were equally good, but correspond to a mixture of species (including the correct one), then the test was ambiguous. The test fails if the match was not conspecific. This test is implemented in TaxonDNA (version 1.6.2, [17]).

Methods used to cluster DNA sequences into MOTUs fall into three categories: (1) tree-based, unsupervised (non-parametric) methods [51]–[53], (2) parametric methods that assume the choice of a threshold in sequence divergence prior to the clustering procedure and that require global sequence alignments [17], (3) alignment-free parametric clustering methods [54], [55]. Although we analyzed our data using all three methods (see Supporting Information S1), the results reported in the main text are based on the alignment-based parametric clustering software TaxonDNA, and on the alignment-free method implemented in *blastclust* (package version 2.2.20 downloaded from ftp://ftp.ncbi.nih.gov/blast/executables/release). The quality of the parametric clustering methods in reference to the morphological taxonomy was assessed by counting, for each threshold sequence distance, the fraction of MOTUs corresponding to more than one taxon (lumping fraction), and the fraction of taxa split into more than one MOTUs (splitting fraction). The lumping fraction should increase with the threshold sequence divergence, while the splitting fraction should decrease. The total number of taxa assigned to a unique MOTU (correct assignment rate) was also reported.

## Results

Depending on the selected marker, we obtained sequences for up to 430 of the sampled individuals, including up to eight markers (a total of 2198 sequences). We obtained high quality sequences in over 90% of the samples for *rpoC1*, *rbcLa*, *rpoB* and *trnL* markers (Table 1). Sequencing success was lower for *psbA-trnH* and *ycf5* (over 80%). A taxonomic bias in sequencing success was detected for *ycf5*, which amplified poorly in the Gentianales (Apocynaceae and Rubiaceae; 7%) and in the Myristicaceae (33%), whereas *rpoB* amplified poorly in the Moraceae (33%). The sequencing success of *matK* was only ∼70%, even after using two different pairs of primers. The lowest sequencing success was obtained with *ITS*, which amplified in only 41% of our samples. The markers varied significantly in mean sequence divergence (Table 1). The highest variability was obtained for *ITS*, followed by *psbA-trnH, trnL* and *matK*.

**Table 1:** Markers for the DNA barcoding of tropical trees.

Marker	Length (bp)	Sequencing success (%)	Nb successfully sequenced individuals	Nb species	Nb genera	Intraspecific divergence	Intraspecific divergence within genus
*rbcLa* *	697	93	368	223	125	0.05%	0.41%
*rpoB* *	475	96	260	173	105	0.04%	0.57%
*rpoC1* *	592	94	430	198	120	0.04%	0.23%
*ycf5* *	276	88	230	155	93	0.18%	0.98%
*matK* *	879	68	182	132	81	0.02%	0.65%
*psbA-trnH* †	264–792	89	369	213	117	0.59%	6.26%
*trnL* †	326–681	93	254	158	88	0.14%	1.06%
*ITS* ‡	488–750	41	105+24†	75+22^a^	43+7^a^	1.73%	6.23%

Eight DNA markers were tested across 49 angiosperm families.

* : coding plastid DNA sequences;

† : non-coding plastid DNA spacers;

‡ : nuclear ribosomal region. Mean intraspecific sequence divergence and interspecific within genus sequence divergence (in %).

a representatives of the sampled species included in the analysis and downloaded from GenBank.

**Figure 1:**
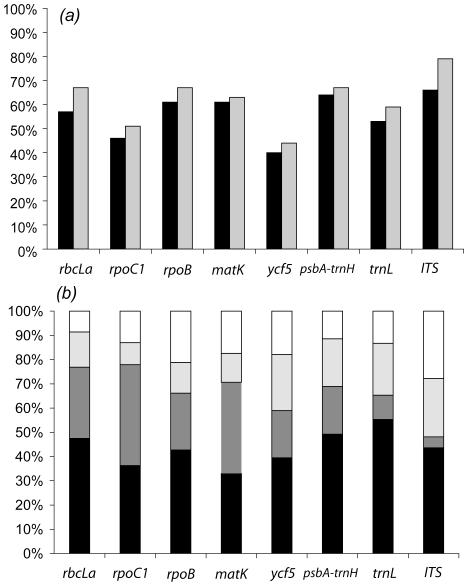
Comparison of DNA barcode performance. Panel (a): Percentage of monophyletic species (black bars) and excluding the Sapotaceae and Laureae (grey bars) using the eight tested markers (see Table 2). Panel (b): Fraction of sequences correctly (black), ambiguously (dark grey), and incorrectly (light grey) assigned to species. Some sequences could not be assigned when their sequence diverged too much from the other species (Table S3).

We assessed the number of monophyletic species recovered in the tree reconstructions for each marker (Fig. 1a). We found little difference between the two methods of phylogenetic tree reconstruction (NJ and ML); and Table 2 reports only the results obtained with the maximum likelihood phylogenetic reconstruction algorithm. When considering all species, the best marker was *psbA-trnH*, which recovered 64% of monophyletic species, followed by *matK*, *rpoB*, and *rbcLa* (Table 2). The poorest performance was obtained with *ycf5* (40%) and *rpoC1* (46%). Ignoring the Sapotaceae and Laureae, the three markers, *psbA-trnH*, *rpoB*, and *rbcLa*, had a similar performance (67%). *ITS* had an excellent performance in recovering monophyletic species, but this represents a biased sample, as we could amplify ITS for less than half of the individuals. Using *rbcLa* or *psbA-trnH*, 77% of the genera were found to be well-supported, while with *ycf5* and *trnL*, this percentage dropped to 63%. The ‘best close match’ test as implemented in TaxonDNA yielded comparable results (Fig. 1b, Table 2). The rates of correct assignment of a randomly selected sequence was maximal for *psbA-trnH* (55%), followed by *trnL* (49%), and *rbcL* (48%). These low values reflect the fact that a large number of sequences were included from the Sapotaceae and Laureae, and these yielded ambiguous assignments.

**Table 2:** Percentage of monophyletic species and percentage of monophyletic genera recovered using the eight tested markers.

Marker	Nb tested species	Percent monophyletic species (rank)	Percent monophyletic species* (rank)	Nb genera	Percent monophyletic genera
*psbA-trnH*	*107*	64 (2)	67 (2)	*82*	77
*matK*	*49*	61 (3)	63 (5)	*45*	71
*rpoB*	*72*	61 (3)	67 (2)	*62*	73
*rbcL*	*104*	57 (5)	65 (4)	*81*	77
*trnL*	*87*	53 (6)	59 (6)	*68*	63
*rpoC1*	*79*	46 (7)	51 (7)	*63*	68
*ycf5*	*62*	40 (8)	44 (8)	*56*	63
*ITS*	*32*	66 (1)	79 (1)	*26*	73

* Excluding Sapotaceae & Laureae.

All eight markers could not be sequenced for exactly the same individuals. Hence, the markers were also compared two by two, based on shared individuals only. This pairwise test of the markers yielded results consistent with the previous analyses (Table S4). In addition, we tested whether combining two markers into a single barcode to discriminate species did increased the performance of the tested markers, and found that this did not greatly improve the overall performance in comparison with single markers (Table S4).

We then tested the performance of each marker in clustering data into MOTUs. With coding cpDNA markers, fewer MOTUs were found than the real number of taxa in our sample (Table 3). Comparing the accuracy of assignment into MOTUs, we used the ‘cluster’ option of TaxonDNA, and found that TaxonDNA returned a mean correct assignment rate of 62% at 0.1% sequence divergence (Table 3, including coding plastid markers and *trnL*). Blastclust provided slightly better results than TaxonDNA both in terms of overall number of MOTUs, and correct assignment rate (Table 3). With blastclust, the rate of correct assignment varied from 80.2% for *ITS* to 53% for *rpoC1* (mean 65.5%). Irrespective of the clustering algorithm, the best rate of correct assignment was obtained for *ITS* followed by *matK*, *psbA-trnH*, *rpoB*, *rbcL* and *trnL*. The worst rate of correct species-level assignment was consistently obtained by *rpoC1*.

**Table 3:** Number of clusters recovered using the tested markers, and with two parametric methods.

Marker	Number of species	TaxonDNA 0.1%	TaxonDNA 0.5%	TaxonDNA 1%	TaxonDNA 3%	TaxonDNA *Rank*
*rbcL*	*223*	187 (60)	113 (34)	93 (26)	47 (10)	*5*
*rpoB*	*173*	145 (60)	99 (43)	85 (34)	52 (15)	*5*
*rpoC1*	*198*	154 (52)	106 (35)	81 (23)	35 (8)	*8*
*ycf5*	*155*	131 (60)	98 (42)	84 (33)	57 (16)	*5*
*matK*	*132*	119 (76)	83 (44)	72 (39)	53 (21)	*2*
*psbA-trnH*	*212*	242 (63)	219 (60)	209 (58)	160 (47)	*3*
*trnL*	*158*	144 (62)	100 (44)	81 (35)	58 (22)	*4*
*ITS*	*101*	114 (73)	104 (77)	94 (70)	76 (52)	*1*
		blastclust 0.1%	blastclust 0.5%	blastclust 1%	blastclust 3%	blastclust Rank
*rbcL*	*223*	186 (62)	114 (34)	95 (25)	48 (17)	6
*rpoB*	*173*	153 (64)	105 (44)	88 (36)	56 (17)	4
*rpoC1*	*198*	154 (53)	107 (36)	82 (23)	36 (8)	8
*ycf5*	*155*	129 (60)	97 (43)	83 (34)	53 (15)	7
*matK*	*132*	118 (75)	84 (44)	78 (40)	61 (24)	1
*psbA-trnH*	*212*	265 (65)	238 (66)	224 (61)	186 (48)	3
*trnL*	*158*	146 (63)	115 (54)	95 (42)	78 (32)	5
*ITS*	*101*	118 (72)	106 (80)	100 (78)	80 (55)	2

TaxonDNA is an alignment-based method based on sequence distance matrices, and blastclust is a method based on blast similarity scores of unaligned sequences. Percentage of correct assignment of a taxon to a MOTU (in parentheses). Given the length of the sequences (<1000 bp), 0.1% generally corresponds to less than 1 bp substitution.

At the genus level, coding chloroplast DNA markers were useful to assign clusters to the correct genus (Fig. S1). For instance, *rpoC1* and *rbcL* reached the best rate of correct genus-level assignment at about 1% in sequence divergence (Fig. S1).

Finally, we attempted to identify tropical saplings by DNA barcoding. First, we clustered the saplings together using *psbA-trnH*, and we then attempted to assign the clusters to recognized species using *psbA-trnH* combined with another marker with a slower rate of molecular evolution (*rpoC1*). This last marker was chosen at the time of the study because it had the highest amplification success. By clustering the *psbA-trnH* sequences, we could define 130 MOTUs (assuming a 1% threshold in sequence divergence, see Table 3). Combining this information with the *rpoC1* marker, we were able to assign 32% of the individuals to a known species, and 25% to a known genus. Lianas and shrubs were quite abundant in the sapling layer, and these lack representatives in our reference database. Restricting our sample to the 152 juveniles of tree species, and based on DNA barcodes only, we detected 86 MOTUs, and we were able to assign 46% of the individuals to a known species, and 29% to a known genus. Finally, combining the morphological and molecular data, we could identify 59% of the individuals to the species, and 37% to the genus. The remaining 4% of the individuals were at least identified to the family level.

## Discussion

We examined whether plant DNA barcoding candidates matched taxonomic species delimitations in a large plant biodiversity survey of an Amazonian forest. Our working assumption was that the rate of species discrimination would exceed 72%, as recently found by the CBoL Plant Working Group [28]. In principle, by restricting the scope of the reference database to species known to occur in a specific habitat or region, a much greater degree of discrimination should be possible, since not all close relatives of a given species occur in the area under study [56]. We collected representatives of truly co-occurring species in order to provide a robust test of *in situ* applications of DNA barcodes. Using a large dataset, all attached to a voucher specimen, we were able to show that correct matching between barcodes and taxonomic species did not exceed 70%. Failure to reach a higher rate of species discrimination was due to the low plastid sequence variation in a few species-rich clades.

We confirmed that the markers *rpoC1*, *rbcLa*, *trnL* and to a lesser extent *rpoB*, could all be sequenced easily from leaf or cambium tissue. Being able to extract DNA directly from the cambium is important because it will prove useful in routine tropical forestry monitoring programs. The other markers showed a lower performance either because they failed in some groups or because they showed a low overall sequencing success. For instance, *matK* could be sequenced in only 68% of our samples, using two primer pairs. CBoL has reported a sequencing success of 90% for the *matK* region [28]. This difference could be explained either by the choice of several combination of primers. Fazekas *et al.* (2008) did report a 88% sequencing success for this marker, but they also emphasized that they had used up to 10 primer pairs, entailing a ‘considerable effort’ [57]. The second option is to use a more sophisticated chemistry at the amplification stage. Ford *et al.* (2009) reported a 85% success for *matK* using a combination of standard and nested multiplexed-tandem PCR (MT-PCR) [27]. The additional cost of testing a large number of primer combinations or of implementing non-standard PCR methods should be considered when implementing a DNA barcode project.

Despite much effort, *ITS* does not seem to compete as a universal DNA barcode for tropical forest inventories given the limited sequencing success observed in this study. Yet, *ITS* could be helpful in the identification of species in some particular target groups, such as the Sapotaceae (unpublished results). Of all coding plastid markers, *ycf5* had consistently the worst performance as a DNA barcode, followed by *rpoC1*. According to the test of monophyly, *matK* and *rpoB* were good barcodes, but not according to the ‘best close match’ test. The *rbcL* marker was intermediate in both tests, but it is both easily sequenced, and well-represented in existing sequence repositories, and the consensus for this marker appears natural [28]. The marker *matK* has been found to provide valuable information in selected groups of plants (genus *Compsoneura*, [30]; Orchidaceae [26],). However, because obtaining sequences for this marker from field-collected plant tissue remains challenging, we suspect that it will be difficult to implement large-scale barcoding projects based on *matK* (see also [27] for a thoughtful discussion). The *trnL^UAA^* intron ranked second in the ‘best close match’ test, and fifth in the monophyly test and in the clustering test (Table 3). It was twice as variable as *rbcL*a, and its variability was comparable to *matK*, but it is much easier to sequence. Hence, it remains an interesting option for barcoding projects [25]. Indeed, the only ecological application of the plant DNA barcoding program thus far is the study of Jurado-Riviera *et al.* (2009), who have used the *trnL* intron to explore the diet of leaf beetles in the Chrysomelinae subfamily [58]. Finally, the use of the *psbA-trnH* marker has been much criticized because it is prone to reads error at the sequencing stage [43]. Yet, in our study, *psbA-trnH* had the best performance as a DNA barcode, ranking first in both monophyly and ‘best close match’ tests, and being universally amplifiable.

Irrespective of the test or of the marker, a remarkable fact is that none of the rates of correct identification exceeded 70%. Part of this limited performance is due to the plant DNA barcoding strategy itself. Most of the markers proposed thus far are located in the chloroplast genome, and as such they do not evolve independently. Species-rich genera, the ones that would benefit the most from molecular identification techniques (*Pouteria*, *Inga*, *Eschweilera*, *Ocotea*) showed little to no variation in the plastid markers. Also, many of our botanical identifications were based on sterile morphological characters, like in all other tropical tree biodiversity surveys. While each single individual had a voucher, which was compared to a reference collection, closely related species often cannot be distinguished based on sterile morphology alone. For example, this is the case of *Trichilia cipo/T. pallida*, *Eschweilera coriacea/E. pedicellata,* and several species in genus *Ocotea*, to cite but a few. One different but equally important problem is that several important tropical tree families are still lacking a comprehensive systematic treatment. For instance, recent work on the Lecythidaceae based on morphology and molecular data showed that several generic delimitations needed to be re-circumscribed [59]. Likewise, large genera such as *Pouteria* are probably not monophyletic [49]. Thus it remains critical for future DNA barcoding projects to keep improving existing repositories through fieldwork and descriptive taxonomy [15].

We used our dataset as a benchmark to assess the performance of several statistical methods to cluster sequences into molecular operational taxonomic units. Both TaxonDNA performed well with all of our markers, and the alignment-free method (blastclust) compares well with TaxonDNA. These methods may be scaled up to very large datasets. This is of considerable current interest, with the development of high-throughput sequencing technologies [60], [61]. These approaches should be of considerable help in accelerating the pace of ecological research and biodiversity monitoring [62].

So far we have ignored the fact that the markers may display a high level of intraspecific geographical structure [63], [64]. To truly test the performance of a putative DNA barcode, it will be essential to sample widely scattered populations for each species to assess the hypothesis that a locally defined reference of DNA barcodes does characterize a species throughout its distributional range. To our knowledge this test has not been performed yet.

It has been argued that plant DNA barcodes could be especially useful to identify juvenile individuals, and plant debris [29]. Here, we tested this idea for the first time, using a two-tiered approach: we first clustered the individuals into MOTUs using the most variable marker *psbA-trnH*. We then assigned the MOTUs to known taxonomic categories using the reference database we had constructed for trees. This enabled us to identify 86 MOTUs within a sample of ca. 152 tree saplings, 96% of which could be identified to the species or at least to the genus. Thus, DNA barcoding does show much potential for accurate identification of species at life stages which have been particularly difficult to investigate using morphological identification only. The coding plastid markers were often not variable enough to identify species. However, they efficiently assigned individuals to higher taxonomic ranks. Even though this differs from the stated goal of DNA barcoding – assigning individuals to species –, it will have important implications for ecological applications, such as tropical plant diversity surveys [11], [12], [65].

## Supporting Information

Supporting Information S1 Additional information on sequence clustering methods
(0.05 MB DOC)

Table S1 List of the sampled individuals with taxonomic identification and accession code. In the last eight columns, the GenBank accession numbers are reported.
(0.15 MB XLS)

Table S2 Primers and PCR conditions for the eight markers tested in the study
(0.06 MB DOC)

Table S3 Test of the DNA markers performance in retrieving the correct species. The option ‘best close match’ of TaxonDNA was used for the eight markers. The ranking of the markers was done according to the rate of correct species assignment in the ‘best close match’ test.
(0.04 MB DOC)

Table S4 Pairwise comparison of the markers to the samples for which both sequences are available. Reported is the percentage of best close match as reported in TaxonDNA for the two markers independently, and also for the combined markers. The rate of correct assignment was less than 50% in most of the cases, and combining two markers did not improve much the rate of correct assignment (+14% on average).
(0.08 MB DOC)

Figure S1 Types of error in the parametric assignment of sequences to MOTUs. Left panel: Error made during the construction of species-level MOTUs. Two types of errors are reported as a function of sequence divergence: splitting of valid taxa into two or more clusters (splitting fraction: squares), and lumping of two or more valid taxa into the same cluster (lumping fraction: circles). Right panel: same as left panel, but using genus-level MOTUs, as the reference taxonomic level.
(3.93 MB TIF)

## References

[1] Smith N, Mori SA, Henderson A, Stevenson DW, Heald SV (2004). Flowering Plants of the Neotropics..

[2] Laurance WF, Nascimento HEM, Laurance SG, Andrade A, Ribeiro JELS (2006). Rapid decay of tree-community composition in Amazonian forest fragments.. Proc Natl Acad Sci USA.

[3] Malhi Y, Roberts JT, Betts RA, Killeen TJ, Li W (2008). Climate change, deforestation, and the fate of the Amazon.. Science.

[4] Hubbell SP, He F, Condit R, Borda-de-Agua L, Kellner J (2008). How many tree species are there in the Amazon, and how many of them will go extinct?. Proc Natl Acad Sci USA.

[5] Balmford A, Bruner A, Cooper P, Costanza R, Farber S (2002). Economic reasons for conserving wild nature.. Science.

[6] Brooks TM, Mittermeier RA, da Fonseca GAB, Gerlach J, Hoffmann M (2006). Global biodiversity conservation priorities.. Science.

[7] Gentry AH (1988). Changes in plant community diversity and floristic composition on environmental and geographical gradients Ann Mo Bot Gard.

[8] Pitman NCA, Terborgh JW, Silman MR, Nuñez P, Neill DA (2001). Dominance and distribution of tree species in upper Amazonian terra firme forests.. Ecology.

[9] Condit R, Pitman N, Leigh EG, Chave J, Terborgh J (2002). Beta diversity in tropical forest trees.. Science.

[10] Tuomisto H, Ruokolainen K, Yli-Halla M (2003). Dispersal, environment, and floristic variation in western Amazonian forests.. Science.

[11] ter Steege H, Pitman NCA, Phillips OL, Chave J, Sabatier D (2006). Continental-scale patterns of canopy tree composition and function across Amazonia.. Nature.

[12] Pitman NCA, Mogollon H, Davila N, Rios M, Garcia-Villacorta R (2008). Tree community change across 700 km of lowland Amazonian forest from the Andean foothills to Brazil.. Biotropica.

[13] Hebert PDN, Cywinska A, Ball SR, de Waard JR (2003). Biological identifications through DNA barcodes.. Proc R Soc B.

[14] Hebert PDN, Stoeckle MY, Zemlack TS, Francis CM (2004). Identification of birds through DNA barcodes.. PLoS Biol.

[15] Moritz C, Cicero C (2004). DNA barcoding: promise and pitfalls.. PLoS Biol.

[16] Floyd R, Abebe E, Papert A, Blaxter M (2002). Molecular barcodes for soil nematode identification.. Mol Ecol.

[17] Meier R, Shiyang K, Vaidya G, Ng PKL (2006). DNA barcoding and taxonomy in Diptera: a tale of high intraspecific variability and low identification success.. Syst Biol.

[18] Hickerson MJ, Meyer CP, Moritz C (2006). DNA barcoding will often fail to discover new animal species over broad parameter space.. Syst Biol.

[19] Elias M, Hill RI, Willmott KR, Dasmahapatra KK, Brower AVZ (2007). Limited performance of DNA barcoding in a diverse community of tropical butterflies.. Proc R Soc B.

[20] Savolainen V, Cowan RS, Vogler AP, Roderick GK, Lane R (2005). Towards writing the encyclopaedia of life: an introduction to DNA barcoding.. Phil Trans Roy Soc B.

[21] Chase MW, Salamin N, Wilkinson M, Dunwell JM, Kesanakurthi RP (2005). Land plants and DNA barcodes: short-term and long-term goals.. Phil Trans Roy Soc B.

[22] KressWJ, Wurdack KJ, Zimmer EA, Weigt LA, Janzen DH (2005). Use of DNA barcodes to identify flowering plants.. Proc Natl Acad Sci USA.

[23] Shaw J, Lickey EB, Schilling EE, Small RL (2007). Comparison of whole chloroplast genome sequences to choose noncoding regions for phylogenetic studies in Angiosperms: the tortoise and the hare III.. Am J Bot.

[24] Kress WJ, Erickson DL (2007). A two-locus global DNA barcode for land plants: the coding rbcL gene complements the non-coding trnH-psbA spacer region PLoS ONE.

[25] Taberlet P, Coissac E, Pompanon F, Gielly L, Miquel C (2007). Power and limitations of the chloroplast trnL (UAA) intron for plant DNA barcoding.. Nucl Acids Res.

[26] Lahaye R, van der Bank M, Bogarin D, Warner J, Pupulin F (2008). DNA barcoding the floras of biodiversity hotspots Proc Natl Acad Sci USA.

[27] Ford CS, Ayres KL, Toomey N, Haider N, van Alphen Stahl J (2009). Selection of candidate coding DNA barcoding regions for use on land plants.. Bot J Linn Soc.

[28] CBoL Plant Working Group (2009). A DNA barcode for land plants.. Proc Natl Acad Sci USA.

[29] Chase MW, Cowan RS, Hollingsworth PM, van der Berg C, Madriñan S (2007). A proposal for a standardised protocol to barcode all land plants.. Taxon.

[30] Newmaster SG, Fazekas AJ, Steeves RAD, Janovec J (2008). Testing candidate plant barcode regions in the Myristicaceae.. Mol Ecol Res.

[31] Coley PD, Barone JA (1996). Herbivory and plant defenses in tropical forests.. Annu Rev Ecol Syst.

[32] Friar EA (2005). Isolation of DNA from plants with large amounts of secondary metabolites..

[33] Linder HP (2008). Plant species radiations: where, when, why?. Phil Trans Roy Soc B.

[34] Richardson JE, Pennington RT, Pennington TD, Hollingsworth PM (2001). Rapid diversification of a species-rich genus of Neotropical rainforest trees.. Science.

[35] Hollingsworth ML, Clark A, Forrest LL, Richardson J, Pennington RT (2009). Selecting barcoding loci for plants: evaluation of seven candidate loci with species-level sampling in three divergent groups of land plants.. Mol Ecol Res.

[36] Couvreur TLP, Chatrou LW, Sosef MSM, Richardson JE (2008). Molecular phylogenetics reveal multiple tertiary vicariance of the African rain forest trees.. BMC Biol.

[37] Smith SA, Donoghue MJ (2008). Rates of molecular evolution are linked to life history in flowering plants.. Science.

[38] Starr JF, Naczi RFC, Chouinart BN (2009). Plant DNA barcodes and species resolution in sedges (Carex, Cyperaceae).. Mol Ecol Res.

[39] Bongers F, Charles-Dominique P, Forget P-M, Théry M (2001). Nouragues: Dynamics and Plant-Animal Interactions in a Neotropical Rainforest..

[40] Mori SA, Prance GT (1987). A guide to collecting Lecythidaceae.. Ann Mo Bot Gard.

[41] De Castilho CV, Magnusson WE, Oliveira de Araujo RN, da Costa Pereira E, Salvino de Souza S (2006). The use of French spikes to collect botanical vouchers in permanent plots: evaluation of potential impacts.. Biotropica.

[42] Deguilloux M-F, Pemonge MH, Petit RJ (2002). Novel perspectives in wood certification and forensics: dry wood as a source of chloroplast, mitochondrial and nuclear DNA.. Proc Roy Soc Lond B.

[43] Devey DS, Chase MW, Clarkson JJ (2009). A stuttering start to plant DNA barcoding: microsatellites present a previously overlooked problem in non-coding plastid regions.. Taxon.

[44] Larkin MA, Blackshields G, Brown NP, Chenna R, McGettigan PA (2007). Clustal W and Clustal X version 2.0 Bioinformatics.

[45] Kimura M (1980). A simple method for estimating evolutionary rates of base substitutions through comparative studies of nucleotide sequences.. J Mol Evol.

[46] Tamura K, Dudley J, Nei M, Kumar S (2007). MEGA4: Molecular Evolution Genetics Analysis (MEGA) software version 4.0.. Mol Biol Evol.

[47] Guindon S, Gascuel O (2003). A simple, fast, and accurate algorithm to estimate large phylogenies by maximum likelihood.. Syst Biol.

[48] Anisimova M, Gascuel O (2006). Approximate likelihood-ratio test for branches: a fast, accurate, and powerful alternative.. Syst Biol.

[49] Swenson U, Anderberg AA (2005). Phylogeny, character evolution, and classification of Sapotaceae (Ericales).. Cladistics.

[50] Chanderbali A, van der Werff H, Renner SS (2001). Phylogeny and historical biogeography of Lauraceae: evidence from the chloroplast and nuclear genomes.. Ann Mo Bot Gard.

[51] Abdo Z, Golding B (2007). A step toward barcoding life: A model-based, decision-theoretic method to assign genes to preexisting species groups.. Syst Biol.

[52] Pons J, Barraclough TG, Gomez-Zurita J, Cardoso A, Duran DP (2006). Sequence-based species delimitation for the DNA taxonomy of undescribed insects.. Syst Biol.

[53] Munch K, Boomsma W, Willerslev E, Nielsen R (2008). Fast phylogenetic DNA barcoding.. Phil Trans Roy Soc B.

[54] Blaxter M, Mann J, Chapman T, Thomas F, Whitton C (2005). Defining operational taxonomic units using DNA barcode data.. Phil Trans Roy Soc B.

[55] Little DP, Stevenson DW (2007). A comparison of algorithms for the identification of specimens using DNA barcodes: Examples from gymnosperms.. Cladistics.

[56] Chase MW, Fay MF (2009). Barcoding of plants and fungi.. Science.

[57] Fazekas AJ, Burgess KS, Kesanakurti PR, Graham SW, Newmaster SG (2008). Multiple multilocus DNA barcodes from the plastid genome discriminate plant species equally well.. PLoS ONE.

[58] Jurado-Riviera JA, Vogler AP, Reid CAM, Petitpierre E, Gómez-Zurita J (2009). DNA barcoding insect-host plant associations.. Proc Roy Soc Lond B.

[59] Mori SA, Tsou C-H, Wu C-C, Cronholm B, Anderberg AA (2007). Evolution of Lecythidaceae with an emphasis on the circumscription of neotropical genera: information from combined ndhF and trnL-F sequence data.. Am J Bot.

[60] Ronaghi M, Uhlen M, Nyren P (1998). A sequencing method based on real-time pyrophosphate.. Science.

[61] Margulies M, Egholm M, Altman WE, Attiya S, Bader JS (2005). Genome sequencing in microfabricated high density picolitre reactors.. Nature.

[62] Valentini A, Pompanon F, Taberlet P (2009). DNA barcoding for ecologists.. Trend Ecol Evol.

[63] Petit RJ, Duminil J, Fineschi S, Hampe A, Salvini D (2005). Comparative organization of chloroplast, mitochondrial and nuclear diversity in plant populations.. Mol Ecol.

[64] Dick CW, Hardy OJ, Jones FA, Petit RJ (2008). Spatial scales of pollen and seed-mediated gene flow in tropical rain forest trees Trop Plant Biol.

[65] Kress WJ, Erickson DL (2009). DNA barcoding – a windfall for tropical biology?. Biotropica.

