# Supplementary material for: Identification of Amazonian Trees with DNA Barcodes
Source: PLoS One. 2009 Oct 16;4(10):e7483. doi: 10.1371/journal.pone.0007483 (PMC2759516; doi:10.1371/journal.pone.0007483)
Supplement: Supporting Information S1 — Additional information on sequence clustering methods (0.05 MB DOC) [file pone.0007483.s001.doc]

**Supporting Information S1.** Additional information on sequence clustering methods

*Non-parametric clustering*

To cluster DNA sequences, the first method we used is a non-parametric coalescent-based approach (Pons *et al.* 2006). It assumes that intraspecific and interspecific gene genealogies have different statistical properties and that they may be modeled differently (the former by a Yule model, the latter by a neutral coalescent, see also Nielsen & Matz 2006). It detects species clusters in the tree, which correspond to the evolutionary boundary among species (Pons *et al.* 2006). In our case, the non-parametric clustering worked well only for the *rpoC1* marker, for which we had typically several samples per species. For *rpoC1*, 209 clusters were obtained, close to the real value of 198 taxa. In existing proposals for the construction of DNA barcoding reference databases, each species should have at least three representatives. Hence, this algorithm may yield more consistent results than the ones obtained here. However, for tropical plants, obtaining three representatives per species represents a formidable logistical challenge. We also used Munch et al. (2009) algorithm, but had trouble at the compilation stage.

*Alignment-based parametric clustering*

In addition to TaxonDNA (see Main Text), we also tested DOTUR, a popular distance-based agglomerative clustering algorithm, developed initially for delimiting microbial OTUs based on 16S rDNA sequences (Schloss & Handelsman 2005). Comparing the accuracy of assignment into MOTUs, we found that DOTUR had a poor performance for all the markers. In addition, DOTUR could not be implemented in the most variable markers (*psbA-trnH* and *ITS*). The error in assignment rate (either by incorrectly lumping two species or splitting one species) was very high with this method. We believe that this is because DOTUR cannot handle sequence distance matrix including high pairwise distances. For these reasons, we do not recommend the use of DOTUR in routine DNA barcoding projects.

*Alignment-free parametric clustering*

Alignment-free algorithms were tested using the blastclust software, which clusters unaligned sequences using a single-linkage clustering algorithm based on megablast similarity scores (part of the blast package version 2.2.20 downloaded from ftp://ftp.ncbi.nih.gov/blast/executables/release/). The blastclust algorithm (part of the blast package version 2.2.20 downloaded from ftp://ftp.ncbi.nih.gov/blast/executables/release/) is similar to previously developed software (Parkinson *et al.* 2002; Blaxter *et al.* 2005). It showed a good clustering performance (see Main Text). In both parametric algorithms, we assumed that threshold sequence divergences range from 0.001 to 0.05. Table S3 provides a comparison between TaxonDNA and blastclust.

In addition to blastclust, we also tested FastgroupII, a software used for clustering 16S rDNA sequences (Yu *et al.* 2006, sequence match option), and freely available online (<http://biome.sdsu.edu/fastgroup/>). FastGroupII usually performed slightly worse than blastclust (mean correct rate of assignment of 62% versus 65.5%). In addition, the pairwise matching algorithm used by Yu *et al.* (2006) is unclear. Consequently, we do not recommend the use of FastGroupII in routine DNA barcoding projects.

Blaxter, M., *et al*. 2005 Defining operational taxonomic units using DNA barcode data *Phil. Trans. R. Soc. B* **360**, 1935-1943

Munch, K., Boomsma, W., Willerslev, E. & Nielsen, R. 2008. Fast phylogenetic DNA barcoding. *Phil. Trans. R. Soc. B* **363**, 3997-4002.

Nielsen, R.,& M. V. Matz. 2006 Statistical approaches for DNA barcoding. *Syst. Biol.* **55**,162-169.

Parkinson, J., Guiliano, D. & Blaxter, M. 2002 Making sense of EST sequences by CLOBBing them. BMC *Bioinformatics* **3,** 31

Pons, J. et al. 2006. Sequence-based species delimitation for the DNA taxonomy of undescribed insects. *Syst. Biol.* **55**, 595-609.

Schloss, P. D. & Handelsman, J. 2005 Introducing DOTUR, a computer program for defining operational taxonomic units and estimating species richness. *Appl. Envir. Microbiol.* **71**, 1501-1506.

Yu, Y., Breitbart, M., McNairnie, P. & Rohwer, F. 2006 FastgroupII: a web-based bioinformatics platform for analyses of large 16S rDNA libraries. *BMC Bioinformatics* **7,** 57-xxx.
