# Supplementary material for: Identification of Amazonian Trees with DNA Barcodes
Source: PLoS One. 2009 Oct 16;4(10):e7483. doi: 10.1371/journal.pone.0007483 (PMC2759516; doi:10.1371/journal.pone.0007483)
Supplement: Table S2 — Primers and PCR conditions for the eight markers tested in the study (0.06 MB DOC) [file pone.0007483.s003.doc]

**Table S2.** Primers and PCR conditions for the eight markers tested in the study.

|  |  |  |  |  |
| --- | --- | --- | --- | --- |
| marker | source | primer | Sequence 5’ – 3’ | PCR conditions |
|  |  |  |  |  |
| *rbcLa* | (a) | 1f | ATGTCACCACAAACAGAAAC | 94ºC 1mn  (94ºC 30s 50ºC 40s 72ºC 40s) x35  72ºC 5min |
|  |  | r724 | TCGCATATGTACCTGCAGTAGC |
|  |  |  |  |  |
| *rpoB* | (a) | 1f | AAGTGCATTGTTGGAACTGG | 94ºC 1mn  (94ºC 30s 50ºC 40s 72ºC 40s) x40 72ºC 5min |
|  |  | 4r | GATCCCAGCATCACAATTCC |
|  |  |  |  |  |
| *rpoC1* | (a) | 1f | GTGGATACACTTCTTGATAATGG | 94ºC 1mn  (94ºC 30s 50ºC 40s 72ºC 40s) x40  72ºC 5min |
|  |  | 4r | CCATAAGCATATCTTGAGTTGG |
|  |  |  |  |  |
| *ycf5* | (a) | 1f | GGATTATTAGTCACTCGTTGG | 94ºC 1mn  (94ºC 30s 50ºC 40s 72ºC 40s)x40  72ºC 5min |
|  |  | 4r | CCCAATACCATCATACTTAC |
|  |  |  |  |  |
| *matK* | (a) | 2.1a | ATCCATCTGGAAATCTTAGTTC | 94ºC 1mn  (94ºC 30s 46ºC 40s 72ºC 40s)x40  72ºC 5min |
|  |  | 3.2r | CTTCCTCTGTAAAGAATTC |
|  |  |  |  |  |
| *matK* | (b) | 390f | CGATCTATTCATTCAATATTTC | 94ºC 1mn (94ºC 1mn 48ºC 40s 72ºC 1mn)x35 72ºC 7min |
|  |  | 1326r | TCTAGCACACGAAAGTCGAAGT |
|  |  |  |  |  |
| *trnH-psbA* | (c) | 3f | GTTATGCATGAACGTAATGCTC | 80ºC 5min  (94°C 30s 56ºC 40s 72ºC 1min)x35  72ºC 10min |
|  |  | f | CGCGCATGGTGGATTCACAATCC |
|  |  |  |  |  |
| trnL | (d) | c | CGAAATCGGTAGACGCTACG | 95ºC 10mn  (95ºC 30s 50ºC 30s 72ºC 2min)x35 |
|  |  | d | GGGGATAGAGGGACTTGAAC |
|  |  |  |  |  |
| ITS | (e) | 17f | ACGAATTCATGGTCCGGTGAAGTGTTCG | 94ºC 45s  (65ºC 30s 72°C 45s)x9  (94ºC 30s 57ºC 30s 72ºC 45s)x24  72°C 3min |
|  |  | 26r | TAGAATTCCCCGGTTCGCTCGCCGTTAC |
|  |  |  |  |  |

(a) Cowan, R. S., Chase, M. W., Kress, W. J. & Savolainen, V. 2006. 300,000 species to identify: problems, progress, and prospects in DNA bar-coding of land plants. *Taxon* **55**, 61 1–616.

(b) Shaw, J. *et al*. 2005 The tortoise and the hare II: Relative utility of 21 noncoding chloroplast DNA sequences for phylogenetic analysis. *Am. J. Bot*. **92**, 142-166. Cuénoud et al., 2002 Am. J. Bot. 89, 132-144

**(c) Cuénou****d, P., Savolainen, V., Chatrou, L. W., Powell, M., Grayer, R. J. and Chase, M. W. 2002.** Molecular phylogenetics of Caryophyllales based on nuclear 18S rDNA and plastid *rbcL, atpB,* and *matK* DNA sequences. Am. J. Bot. 89, 132-144.

(d) Taberlet, P. *et al.* 2007 Power and limitations of the chloroplast trnL (UAA) intron for plant DNA barcoding *Nucl. Acids Res.* **35,** e17.

(e) Sun, Y., Skinner, D. Z., Liang, G. H. & Hulbert, S. H. 1994. Phylogenetic analysis of *Sorghum* and related taxa using internal transcribed spacers of nuclear ribosomal DNA *Theor. Appl. Gen.* **89**, 26-32
