# Supplementary material for: Identification of Amazonian Trees with DNA Barcodes
Source: PLoS One. 2009 Oct 16;4(10):e7483. doi: 10.1371/journal.pone.0007483 (PMC2759516; doi:10.1371/journal.pone.0007483)
Supplement: Table S3 — Test of the DNA markers performance in retrieving the correct species. The option ‘best close match’ of TaxonDNA was used for the eight markers. The ranking of the markers was done according to the rate of correct species assignment in the ‘best close match’ test. (0.04 MB DOC) [file pone.0007483.s004.doc]

**Table S3.** Test of the DNA markers performance in retrieving the correct species. The option ‘best close match’ of TaxonDNA was used for the eight markers. The ranking of the markers was done according to the rate of correct species assignment in the ‘best close match’ test.

|  |  |  |  |  |  |  |  |
| --- | --- | --- | --- | --- | --- | --- | --- |
| marker | Nb seq. | Correct | Ambiguous | Incorrect | Unassigned | % correct | Rank |
| *rpoC1* | 430 | 156 | 179 | 39 | 56 | 36.3 | 7 |
| *rbcL* | 368 | 161 | 98 | 38 | 71 | 43.8 | 3 |
| *ycf5* | 225 | 72 | 82 | 26 | 38 | 32.0 | 8 |
| *rpoB* | 260 | 107 | 61 | 35 | 57 | 41.2 | 6 |
| *trnL* | 254 | 122 | 52 | 51 | 29 | 48.0 | 2 |
| *psbA-trnH* | 369 | 206 | 37 | 76 | 50 | 55.8 | 1 |
| *matK* | 182 | 77 | 32 | 36 | 37 | 42.3 | 5 |
| *ITS* | 133 | 58 | 6 | 53 | 16 | 43.6 | 4 |
|  |  |  |  |  |  |  |  |
