# Supplementary material for: Identification of Amazonian Trees with DNA Barcodes
Source: PLoS One. 2009 Oct 16;4(10):e7483. doi: 10.1371/journal.pone.0007483 (PMC2759516; doi:10.1371/journal.pone.0007483)
Supplement: Table S4 — Pairwise comparison of the markers to the samples for which both sequences are available. Reported is the percentage of best close match as reported in TaxonDNA for the two markers independently, and also for the combined markers. The rate of correct assignment was less than 50% in most of the cases, and combining two markers did not improve much the rate of correct assignment (+14% on average). (0.08 MB DOC) [file pone.0007483.s005.doc]

**Table S4**. Pairwise comparison of the markers to the samples for which both sequences are available. Reported is the percentage of best close match as reported in TaxonDNA for the two markers independently, and also for the combined markers. The rate of correct assignment was less than 50% in most of the cases, and combining two markers did not improve much the rate of correct assignment (+14% on average).

|  |  |  |  |  |  |  |  |
| --- | --- | --- | --- | --- | --- | --- | --- |
| Number of sequences | marker 1 | Correct assignment (%) | marker 2 | Correct assignment (%) | marker1+ marker2 | Correct assignment (%) | Rank |
| 190 | *rpoB* | **46.3** | *trnL* | **50.5** | *rpoB+trnL* | **53.2** | *rpoB<trnL* |
| 230 | *rpoB* | **41.7** | *psbA-trnH* | **53.5** | *rpoB+psbA-trnH* | **53.0** | *rpoB<psbA-trnH* |
| 239 | *rbcL* | **41.8** | *psbA-trnH* | **55.2** | *rbcL+psbA-trnH* | **52.7** | *rbcL<psbA-trnH* |
| 203 | *psbA-trnH* | **49.8** | *trnL* | **45.8** | *psbA-trnH+trnL* | **52.7** | *psbA-trnH>trnL* |
| 86 | *rbcL* | **31.4** | *ITS* | **46.5** | *rbcL+ITS* | **47.7** | *rbcL<ITS* |
| 202 | *ycf5* | **30.2** | *psbA-trnH* | **48.5** | *ycf5+psbA-trnH* | **47.5** | *ycf5<psbA-trnH* |
| 243 | *rbcL* | **39.5** | *rpoB* | **42.8** | *rbcL+rpoB* | **47.3** | *rbcL<rpoB* |
| 130 | *matK* | **43.1** | *trnL* | **42.3** | *matK+trnL* | **46.9** | *matK=trnL* |
| 75 | *rpoB* | **36.0** | *ITS* | **45.3** | *rpoB+ITS* | **46.7** | *rpoB<ITS* |
| 98 | *ITS* | **48.0** | *trnL* | **37.8** | *ITS+trnL* | **45.9** | *ITS>trnL* |
| 170 | *ycf5* | **32.4** | *trnL* | **46.5** | *ycf5+trnL* | **45.9** | *ycf5<trnL* |
| 172 | *rpoB* | **34.9** | *matK* | **42.4** | *rpoB+matK* | **45.3** | *rpoB<matK* |
| 204 | *rbcL* | **44.1** | *trnL* | **46.6** | *rbcL+trnL* | **44.1** | *rbcL<trnL* |
| 213 | *rpoC1* | **30.0** | *psbA-trnH* | **43.7** | *rpoC1+psbA-trnH* | **43.7** | *rpoC1<psbA-trnH* |
| 179 | *rpoC1* | **34.6** | *trnL* | **43.0** | *rpoC1+trnL* | **43.6** | *rpoC1<trnL* |
| 219 | *rpoB* | **40.2** | *ycf5* | **31.1** | *rpoB+ycf5* | **43.4** | *rpoB>ycf5* |
| 159 | *matK* | **38.4** | *psbA-trnH* | **42.8** | *matK+psbA-trnH* | **42.8** | *matK<psbA-trnH* |
| 85 | *ITS* | **41.2** | *psbA-trnH* | **42.4** | *ITS+psbA-trnH* | **42.4** | *ITS=psbA-trnH* |
| 66 | *ITS* | **40.9** | *ycf5* | **21.2** | *ITS+ycf5* | **40.9** | *ITS>ycf5* |
| 222 | *rbcL* | **36.0** | *rpoC1* | **30.2** | *rbcL+rpoC1* | **40.5** | *rbcL>rpoC1* |
| 215 | *rbcL* | **35.8** | *ycf5* | **31.6** | *rbcL+ycf5* | **40.5** | *rbcL>ycf5* |
| 195 | *rpoC1* | **32.8** | *rpoB* | **36.9** | *rpoC1+rpoB* | **39.0** | *rpoC1<rpoB* |
| 44 | *matK* | **34.1** | *ITS* | **38.6** | *matK+ITS* | **38.6** | *matK<ITS* |
| 149 | *matK* | **35.6** | *ycf5* | **20.8** | *matK+ycf5* | **37.6** | *matK>ycf5* |
| 174 | *rpoC1* | **35.1** | *ycf5* | **28.7** | *rpoC1+ycf5* | **35.6** | *rpoC1>ycf5* |
| 171 | *rbcL* | **31.0** | *matK* | **36.8** | *rbcL+matK* | **32.2** | *rbcL<matK* |
| 129 | *rpoC1* | **24.8** | *matK* | **29.5** | *rpoC1+matK* | **29.5** | *rpoC1<matK* |
| 66 | *rpoC1* | **21.2** | *ITS* | **15.2** | *rpoC1+ITS* | **25.8** | *rpoC1>ITS* |
|  |  |  |  |  |  |  |  |
