# Supplementary figures and images for: Identification of Amazonian Trees with DNA Barcodes
Source: PLoS One. 2009 Oct 16;4(10):e7483. doi: 10.1371/journal.pone.0007483 (PMC2759516; doi:10.1371/journal.pone.0007483)

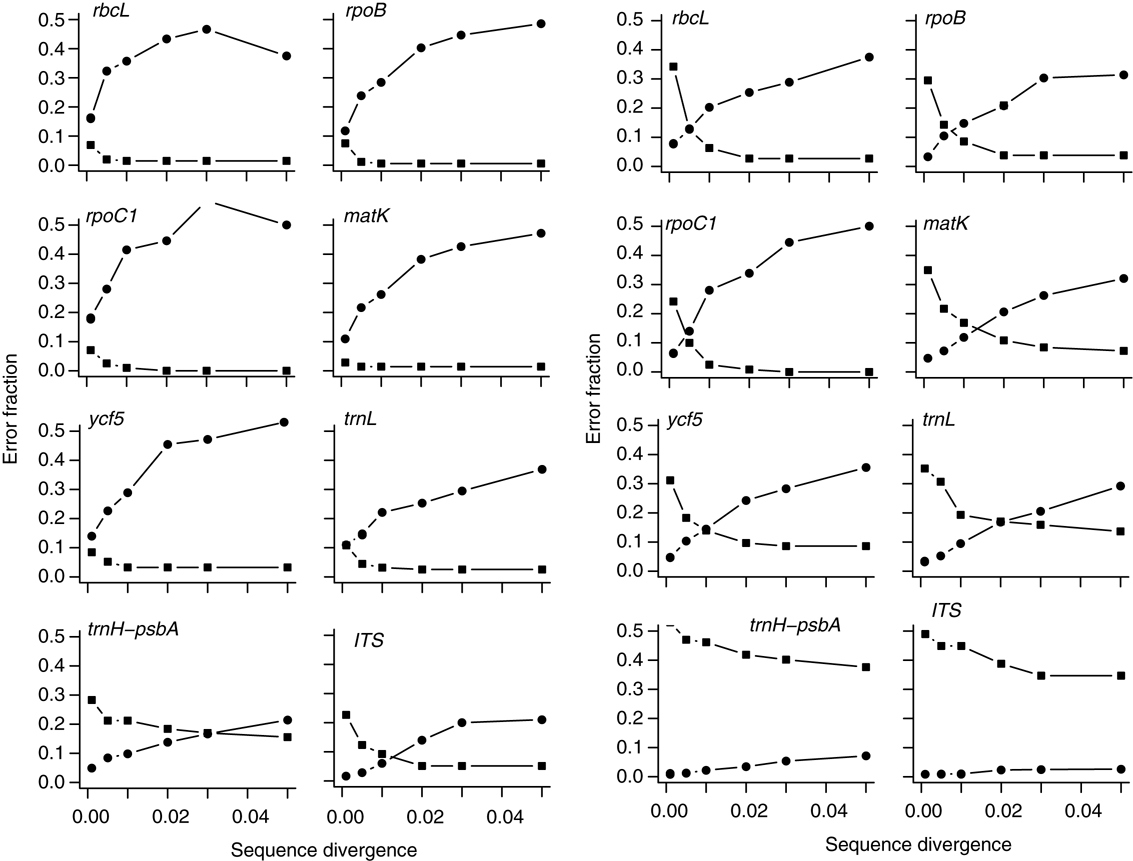

Supplement: Figure S1 — Types of error in the parametric assignment of sequences to MOTUs. Left panel: Error made during the construction of species-level MOTUs. Two types of errors are reported as a function of sequence divergence: splitting of valid taxa into two or more clusters (splitting fraction: squares), and lumping of two or more valid taxa into the same cluster (lumping fraction: circles). Right panel: same as left panel, but using genus-level MOTUs, as the reference taxonomic level. (3.93 MB TIF) [file pone.0007483.s006.tif]
